# Metabolic Response to NAD Depletion across Cell Lines Is Highly Variable

**DOI:** 10.1371/journal.pone.0164166

**Published:** 2016-10-06

**Authors:** Yang Xiao, Mandy Kwong, Anneleen Daemen, Marcia Belvin, Xiaorong Liang, Georgia Hatzivassiliou, Thomas O’Brien

**Affiliations:** 1 Department of Translational Oncology, Genentech Inc. South San Francisco, California, United States of America; 2 Department of Bioinformatics and Computational Biology, Genentech Inc. South San Francisco, California, United States of America; 3 Department of Drug Metabolism and Pharmacokinetics, Genentech Inc. South San Francisco, California, United States of America; University of South Alabama, UNITED STATES

## Abstract

Nicotinamide adenine dinucleotide (NAD) is a cofactor involved in a wide range of cellular metabolic processes and is a key metabolite required for tumor growth. NAMPT, nicotinamide phosphoribosyltransferase, which converts nicotinamide (NAM) to nicotinamide mononucleotide (NMN), the immediate precursor of NAD, is an attractive therapeutic target as inhibition of NAMPT reduces cellular NAD levels and inhibits tumor growth *in vivo*. However, there is limited understanding of the metabolic response to NAD depletion across cancer cell lines and whether all cell lines respond in a uniform manner. To explore this we selected two non-small cell lung carcinoma cell lines that are sensitive to the NAMPT inhibitor GNE-617 (A549, NCI-H1334), one that shows intermediate sensitivity (NCI-H441), and one that is insensitive (LC-KJ). Even though NAD was reduced in all cell lines there was surprising heterogeneity in their metabolic response. Both sensitive cell lines reduced glycolysis and levels of di- and tri-nucleotides and modestly increased oxidative phosphorylation, but they differed in their ability to combat oxidative stress. H1334 cells activated the stress kinase AMPK, whereas A549 cells were unable to activate AMPK as they contain a mutation in LKB1, which prevents activation of AMPK. However, A549 cells increased utilization of the Pentose Phosphate pathway (PPP) and had lower reactive oxygen species (ROS) levels than H1334 cells, indicating that A549 cells are better able to modulate an increase in oxidative stress. Inherent resistance of LC-KJ cells is associated with higher baseline levels of NADPH and a delayed reduction of NAD upon NAMPT inhibition. Our data reveals that cell lines show heterogeneous response to NAD depletion and that the underlying molecular and genetic framework in cells can influence the metabolic response to NAMPT inhibition.

## Introduction

Appropriate regulation of cellular metabolism is critical to sustain cell proliferation and involves a tremendous complexity that includes cross-talk across a variety of metabolic pathways [1]. In particular, the high proliferative capacity of cancer cells requires a constant need for cellular metabolism to support growth and these cells are known to alter metabolic pathways to their advantage, for example, by increasing anaerobic glycolysis [2, 3].

NAD and its derivatives (NADH, NADP, NADPH) serve as cofactors in multiple metabolic pathways regulating the redox status of cells (NAD/NADH ratio), signal transduction (e.g. cofactor for SIRT) and for DNA repair (e.g. cofactor for PARP1)[4]. NAD is generated either by de novo synthesis starting from the essential amino acid tryptophan, or from nicotinamide by the salvage pathway [5]. The rate limiting enzyme in NAD generation is NAMPT, which generates nicotinamide mononucleotide (NMN) by an ATP dependent mechanism that involves the condensation of nicotinamide (NAM) with 5-phosphoribosyl-1-pyrophosphate (PRPP). NMN is the immediate precursor of NAD. Given the many functions of NAD in supporting proliferation, and the observation that tumors consume NAD at a higher rate than normal tissue, inhibition of NAD synthesis has been proposed as an attractive therapeutic strategy. In recent years, several NAMPT inhibitors have been described, (GNE-617 [6]; APO866[7], GMX1778 [8]; reviewed by Montecucco et al [9]) and as expected, inhibiting NAMPT leads to depletion of NAD and tumor growth inhibition both *in vitro* and *in vivo*.

Depletion of NAD in cells has been shown to block glycolysis, increase utilization of the pentose phosphate pathway (PPP) and increase glutaminogenesis [10, 11]. However, there is still a limited understanding of how metabolic effects vary across cell lines with varying sensitivities to NAMPT inhibitors. To profile a broader spectrum of metabolic response to NAD depletion, we assessed the effects of NAD depletion induced by GNE-617 in a panel of four non-small cell lung cancer cell lines, including two cell lines that are sensitive, one that is moderately sensitivity, and one that is insensitive to GNE-617. Our data demonstrate a surprising level of metabolic heterogeneity across cell lines in their responses to NAD depletion. Some of this heterogeneity is likely driven by the genetic profile of each cell line. A549 cells, for example, harbor a mutation in LKB1 and do not activate AMPK in response to an increase in the AMP:ATP ratio. Nevertheless, this study has revealed that the metabolic response to loss of NAD varies greatly across cell lines, and provides insight on why some cell lines may be inherently less sensitivity to inhibition of NAMPT.

## Materials and Methods

### Cell culture and reagents

Cell lines were obtained from American Type Culture Collection (ATCC), expanded, and stored at early passage in a central cell bank at Genentech. Short tandem repeat (STR) profiles were determined for each line using the Promega PowerPlex 16 System. STR profiling was performed once and compared with external STR profiles of cell lines (when available) to determine cell line ancestry. SNP profiles were performed each time new stocks were expanded for cryopreservation. Cell line identity was verified by high-throughput SNP profiling using Fluidigm multiplexed assays. SNPs were selected based on minor allele frequency and presence on commercial genotyping platforms. SNP profiles were compared with SNP calls from available internal and external data (when available) to determine or confirm ancestry. In cases where data were unavailable or cell line ancestry was questionable, DNA or cell lines were repurchased to perform profiling to confirm cell line ancestry.

During the experiments, cells were maintained in RPMI with 10% FBS and 2mM Glutamine. All cell lines were maintained below a passage number of 20. The small molecule inhibitor, GNE-617, was synthesized in-house[6].

Antibodies used in this study included NAMPT (clone 4D5, Cat. No. NBP1-0435; Novus, Littleton, CO; AB_1522075), which was used at a 1:1,000 dilution, Actin (Cat#A5441; Sigma; AB_476744) which was used at a dilution of 1:5,000, GAPDH (Cat. No. 2118, Cell Signaling Technology; AB_1031003) which was used at a dilution of 1:2,000, AMPK (clone 2B7,Cat. No. NBP2-22127; Novus, Littleton, CO), which was used at a dilution of 1:1,000, p-AMPK-T172 (Cat#2535, Cell Signaling Technology) which was used at a dilution of 1:1,000, and G6PD (Clone D5D2, Cat#12263, Cell Signaling Technology) which was used at a dilution of 1:1,000.

### Cell based assays

Cells were treated either with a dose titration of GNE-617, or with 0.2 or 0.4 **μ**M GNE-617 as indicated, and were harvested at various times to measure NAD or ATP levels or for viability. Cellular NAD levels were measured by LC-MS as previously described [12]. ATP levels were measured by CelltiterGlo (Promega) and nuclei content was measured by CyQuant-direct (Life Tech).

For siRNA experiments, an siRNA pool against G6PD (Dharmacon) were transfected into each cell line with RNAiMax (LifeTech, 40 nM oligo, 5 **μ**l RNAiMax per well in 6 well plates) according to manufactors protocol. Twenty-four hours after reverse transfection, cells were split into 1 x 384 well plate and 2 x 6 well plates. Twenty-four hours after plating, compounds were added to the 384 well plates to test for viability (4 day incubation and viability was assessed using CellTiter Glo), and the 6 well plates were collected for Western Blotting (120 hours post-transfection).

For ROS measurements cells were treated with DMSO or GNE-617 for the designated times. ROS levels were detected with the ENZO detection kit (ENZ 51010) and measured according to the manufactors protocol.

### Metabolite analysis

Cells were plated in LUMOX plates (Sarstedt, 946077.331) such that cell density was approximate 2 x 10^6^ per well at the time of collection and DMSO or GNE-617 was added the next day. All samples were collected and processed as previously described by Metanomics Health, and this analysis provided a comprehensive quantitative analysis of each metabolite and included the addition or spike-in of a ^13^C-labeled control into each sample prior to extraction and mass spectrometry analysis, allowing quantitative comparison of metabolite levels across cell lines [13, 14]. A total of 6 replicates were collected for each condition.

### Cellular labeling and flux studies

Cells were plated in 6 well plates such that cell density was approximate 2 x 10^6^ per well at the time of collection. The next day cell medium was replaced with medium containing U-^13^C_6_-glucose (Cambridge Isotope) and unlabeled glucose at 1:1 ratio (final concentration of 5 mM), or U-^13^C_6_-glutamine and unlabeled glutamine at 1:1 ratio (final concentration of 2 mM). GNE-617 or DMSO was added at the same time. Cells were collected 48 hours later with cold extraction solution (Acetonitrile/Methanol/H2O, 4.5/4.5/1). A total of 5 replicates were collected for each condition.

## Results

### Identification of GNE-617 sensitive and resistant NSCLC cells

**Fig 1:**
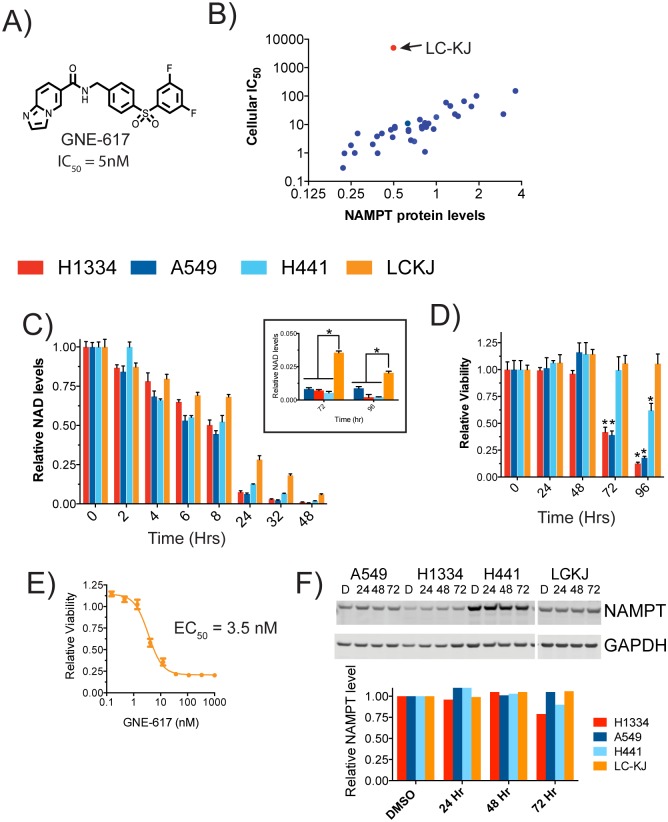
GNE-617 reduces NAD levels in sensitive and resistant cell lines. A) Structure of GNE-617 with its biochemical IC50 for NAMPT. B) Correlation between NAMPT protein levels and sensitivity to GNE-617 (IC50) determined in a 4-day viability assay. IC50 values were previously reported [15]; protein levels were quantified from western blots shown in S1A Fig. Full-length western blots are shown in S2A Fig. C) NAD levels in each of the four cell lines was determined by mass spectrometry at various time after treatment with 0.4 μM GNE-617 (n = 3, ± SD). Insert shows data with a different y-axis scale at 72 and 96 hours. * indicates a p-value of <0.05, and statistical analysis was performed using a Mann Whitney t-test (two-tailed). D) Relative viability of each cell line, as assessed by a CyQuant readout, was determined at various time after treatment with 0.4 μM GNE-617 (n = 3, ± SD). * indicates a p-value of <0.05, and statistical analysis was performed using a Mann Whitney t-test (two-tailed). E) LC-KJ cells were treated with a dose response of GNE-617 and viability determined after 7-days (CyQuant)(n = 3, ± SD). F) NAMPT protein levels were determined in each cell line at the indicated times after exposure to 0.4 μM GNE-617. Full western blots for this image are shown in S2B Fig. Shown below is quantitation of NAMPT levels (relative to GAPDH protein levels) for each time point.

GNE-617 is a potent NAMPT inhibitor with a biochemical IC_50_ of 5 nM (Fig 1A) (compound 58 in [6]). This compound was previously screened across a panel of non-small cell lung carcinoma (NSCLC) cell lines in a 4-day viability assay, which revealed that approximately 50% of all cell lines had an IC_50_ of < 10 nM [15]. However, there was a wide spectrum of sensitivity to GNE-617, with some cell lines, such as LC-KJ, inherently resistant to GNE-617 (IC_50_ >10 **μ**M). Sensitivity of this NSCLC panel of cell lines to GNE-617 was found to inversely correlate with NAMPT mRNA levels, consistent with previous reports [15]. This observation also holds up at the protein level, as we observed that NAMPT protein levels inversely correlated with sensitivity to GNE-617 (Fig 1B, see S1A Fig). However, LC-KJ is a notable exception as this cell line has relative low NAMPT protein levels yet is insensitive to GNE-617 in a 4-day viability assay (Fig 1B). Given this wide range of sensitivity to NAMPT inhibition, we were interested in determining the underlying metabolic dependencies in cells with varying sensitivity to NAMPT inhibition. H1334, A549, H441, and LC-KJ cells lines were chosen as these exhibited a wide range of sensitivity to NAMPT inhibition in a four-day viability assay (S1B Fig).

The kinetics of NAD depletion in response to NAMPT inhibition were examined in each cell line and, consistent with previous reports showing rapid depletion of NAD in sensitive cells in response to NAMPT inhibition [12, 16–19], NAD was significantly reduced in all cell lines within ~48 hours of treatment (Fig 1C). Surprisingly, NAD levels in the inherently resistant LC-KJ cell line were also reduced, although this occurred at a slower rate compared to the other three cell lines. When examined at later times, NAD levels in LC-KJ cells continued to decline even after 96 hours of treatment (Fig 1C, insert). The kinetics of loss of viability was also assessed. As expected, both sensitive cell lines (H1334 and A549) rapidly reduced viability within 72 hours, whereas the less sensitive cell line H441 retained ~50% viability at 96 hours (Fig 1D). Given that LC-KJ cells had significantly reduced NAD levels by 96 hours, we evaluated viability of these cells at a later time point, as loss of cellular viability is not observed until after NAD is depleted by ~>95% [16]. Incubating LC-KJ cells with GNE-617 for 7 days resulted in a significant reduction of viability with an IC_50_ of 0.003 **μ**M (Fig 1E), indicating that sustained inhibition of NAD generation can induce death in these cells.

Given that NAMPT protein levels anti-correlate with sensitivity to NAMPT inhibition, one hypothesis was that LC-KJ cells rapidly increased NAMPT levels in response to GNE-617. To assess if levels of NAMPT changed in these cell lines after treatment with GNE-617, NAMPT protein levels in all four cell lines were determined at 24, 48 and 72 hours following exposure to GNE-617. No obvious increase of NAMPT was observed in any of the four cell lines (Fig 1F). Thus, decreased sensitivity of LC-KJ cells to GNE-617 is not due to a transient increase in NAMPT protein levels.

### Global metabolic effects are observed in cells after NAMPT inhibition

To determine the metabolic consequences of NAD depletion across cell lines, we comprehensively profiled 97 different metabolites in each cell line at three different time-points (24, 48 and 72 hours) by mass spectrometry.

**Fig 2:**
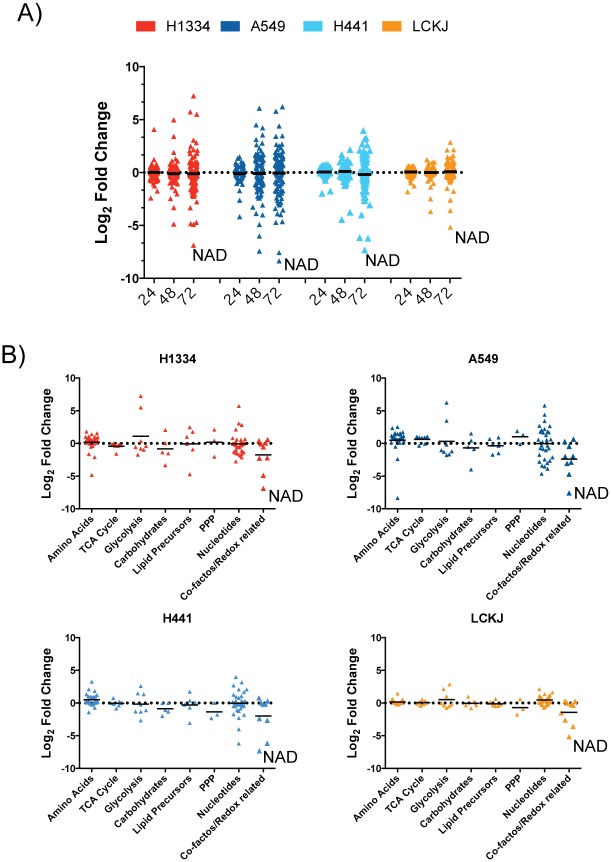
Reduction of NAD results in large metabolic changes. A) Levels of 97 different metabolites were assessed in each cell line as described in the methods section at 24, 48 or 72 hours after treatment with 0.4 μM GNE-617 (n = 5, average of each metabolite is shown). Shown is the log2-fold change for the level of each metabolite relative to its level in untreated cells. B) Changes in different categories of metabolites at 72 hours in each cell line, as determined in panel A.

We observed large global effects due to NAD depletion in each cell, and these effects increased over time (Fig 2A; S1 Table). The metabolite that is reduced to the largest extent in each cell line at 72 hours is NAD. The metabolic profile of the more resistant LC-KJ cells changed after 72 hours of treatment and many of these changes are associated with metabolites involved in NAD biosynthesis (Fig 2B). Moreover, after 72 hours, each cell line shows similar metabolic profiles for the pathways that are modulated. For example, there is a general increase in metabolites associated with glycolysis, there is perturbation of nucleotide biosynthesis, and there is reduction in steady state levels of co-factors (Fig 2B).

### A549 cells preferentially rely on the PPP to respond to NAD depletion

**Fig 3:**
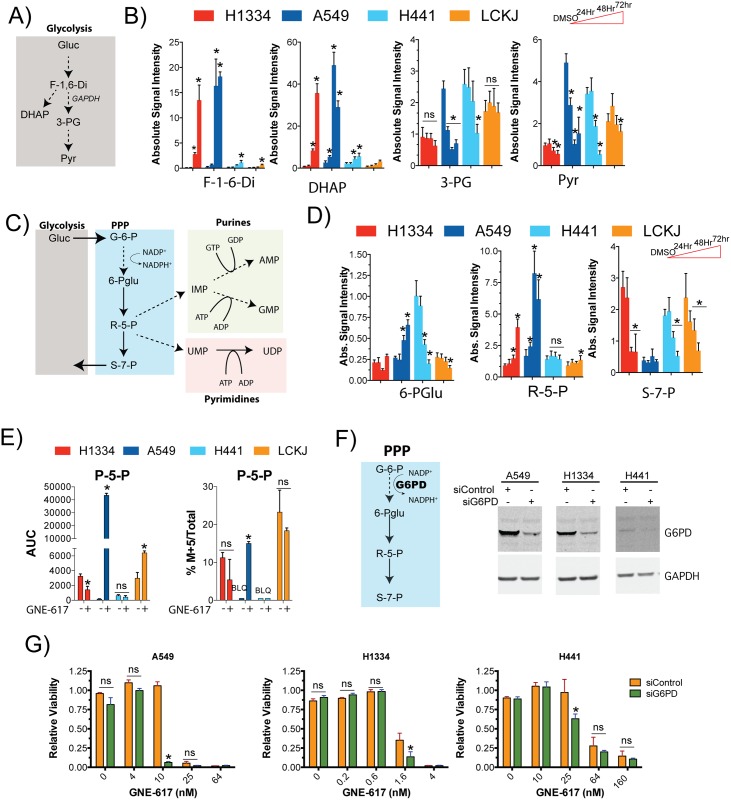
A549 cells increase reliance on the PPP following NAMPT inhibition. Schematic of key metabolites in glycolysis. B) Cells were treated with 0.4 μM GNE-617 and levels of each metabolite was determined as described in Fig 2A (Ave ± SD, n = 5). * indicates a p-value of <0.05, and statistical analysis was performed using a Mann Whitney t-test (two-tailed); “ns” indicates not-statistically significant. C) Schematic of key metabolites in the PPP and in nucleotide biosynthesis. D) Cells were treated as in part B and levels of each metabolite was determined as described in Fig 2A (Ave ± SD, n = 5). * indicates a p-value of <0.05, and statistical analysis was performed using a Mann Whitney t-test (two-tailed); “ns” indicates not-statistically significant. E) Each cell line was exposed to 0.4 μM GNE-617 for 48 hours and labeled with [13C-6]glucose. Levels of newly synthesized pentose-5-phosphate were determined relative to total metabolite levels (Ave ± SD, n = 3). * indicates a p-value of <0.05, and statistical analysis was performed using a Mann Whitney t-test (two-tailed); “ns” indicates not-statistically significant. F) Schematic of the PPP with the step catalyzed by G6PD. Cells were treated with siRNA directed against G6PD and protein levels were examined after 5 days. Full-length western blots are shown in S2C Fig. G) Cells were treated either with siControl or siRNA directed against G6PD for 24 hours, and then treated with different concentrations of GNE-617 for an additional 4 days at which point cell viability was assessed (Ave ± SD, n = 3). * indicates a p-value of <0.05, and statistical analysis was performed using a Mann Whitney t-test (two-tailed); “ns” indicates not-statistically significant.

Previous reports have shown that NAD depletion results in a block of the conversion of glyceraldehyde-3-phosphate to 1,3-bisphosphoglycerate, a key step in glycolysis that is catalyzed by the NAD utilizing enzyme glyceraldehyde-3-phosphate dehydrogenase (GAPDH) (See Fig 3A) [10, 20]. Consistent with this, we observed an increase in metabolites upstream of GAPDH (fructose 1-6-diphosphate and DHAP) and a decrease of downstream metabolites such as 3-phosphoglycerate and pyruvate in a time dependent manner in the two sensitive cell lines, A549 and H1334 (Fig 3B). Levels of these metabolites did not significantly change in LC-KJ cells.

One potential consequence of a block in glycolysis is for cells to shunt excess glycolytic metabolites into the PPP (Fig 3C). Cells use the PPP to generate NADPH, the primary metabolite that provides reducing potential for the cell, and ribose-5-phosphate (R-5-P), the precursor for purine and pyrimidine biosynthesis. A549 cells and, to a lesser extent, H1334 cells compensate for excess glycolytic intermediates by redirecting carbon flow from glycolysis to the PPP, which is evident by an increase in 6-phosphogluconic acid (6-PGlu) and R-5-P within ~48 hours following NAMPT inhibition (Fig 3D). Interestingly, no increase in levels of sedoheptulose-7-phosphate (S-7-P) were observed (Fig 3D), suggesting that the excess carbon may be shunted towards nucleotide biosynthesis rather than back into glycolysis via the non-oxidative phase of the PPP. In contrast, H441 cells did not show an increase in these metabolites rather, levels of 6-PGlu and S-7-P were reduced at later time points. The only notable change in LC-KJ cells was reduction of S-7-P levels by 72 hours, suggesting that these cells do not dramatically decrease glycolysis nor use the PPP in response to NAMPT inhibition.

A [^13^C-6]-glucose labeling experiment was performed to directly examine carbon flow through the PPP pathway in each cell line. Baseline levels of pentose-5-phosphate (P-5-P) were low in both H441 and A549 cells (Fig 3E). Yet, following treatment with GNE-617, there was a large increase in carbon flow into the PPP in A549 cells, as evident by an increase in both total and the M+5 isotopomer of P-5-P, which was consistent with our steady state measurements (Fig 3D). No increase in the flow of carbon to PPP was observed in H441 and H1334 cells.

If A549 cells preferentially rely on the PPP after NAMPT inhibition, then they should be sensitive to inhibition of this pathway. To test this glucose-6-phosphate dehydrogenase (G6PD), the enzyme responsible for the conversion of glucose-6-phosphate to 6-phosphoguconate in the PPP, was depleted by siRNA in A549, H1334 and H441 cells (Fig 3F). Levels of G6PD were reduced in all three cell lines following G6PD siRNA treatment, although baseline levels in H441 cells were significantly lower than in the other two cell lines. Depletion of G6PD sensitized A549 cells to GNE-617, emphasizing the importance of the PPP to these cells following NAMPT inhibition. In contrast, H1334 and H441 cells only displayed low levels of enhanced sensitivity, indicating that these cell lines have less dependence on this pathway compared to A549 cells.

### Cells sensitive to NAMPT inhibition have defects in the ability to generate diphosphorylated- and triphosphorylated-nucleotides

**Fig 4:**
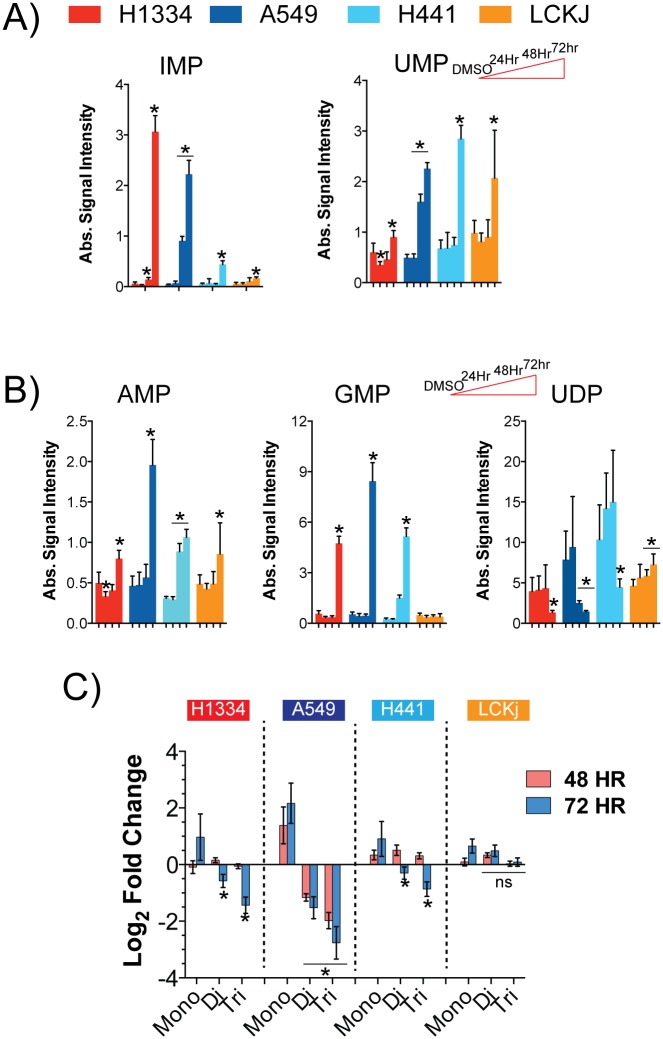
Sensitive cell lines show defects in purine and pyrimidine biosynthesis. A-B) Cells were treated with 0.4 μM GNE-617 and levels of UMP and IMP (A), GMP and UDP (B) were determined as described in Fig 2A (Ave ± SD, n = 5). * indicates a p-value of <0.05, and statistical analysis was performed using a Mann Whitney t-test (two-tailed); “ns” indicates not-statistically significant. C) Level of mono-nucleotides (n = 8), diphosphorylated-nucleotides (n = 7) and triphosphorylated-nucleotides (n = 7) in cells were determined following treatment with 0.4 μM GNE-617 (shown is the average and SD for each group). * indicates a p-value of <0.05, and statistical analysis was performed using a Mann Whitney t-test (two-tailed); “ns” indicates not-statistically significant.

A function of the P-5-P produced by the PPP is to serve as a precursor for the generation of purines and pyrimidines. Both sensitive cell lines, A549 and H1344, increased levels of IMP and UMP (Fig 4A) and their downstream metabolites AMP and GMP after treatment with GNE-617 (Fig 4B). However, UDP levels were decreased in both the sensitive and the moderately sensitive (H441) cell lines. As cells have lower NAD and ATP levels in response to NAMPT inhibition, then one expectation is that cells would have greater difficulty generating both diphosphorylated- and triphosphorylated-nucleotides. Consistent with this prediction, a noticeable drop in levels of diphosphorylated- and triphosphorylated-nucleotides was found in sensitive and moderately sensitive cell lines within 72 hours (Fig 4C). The increase in monophosphate nucleotides may therefore be due to the inability of cells to convert mono-phosphates into diphosphorylated- and triphosphorylated-nucleotides.

Even though we did not observe an increase in carbon flow through the PPP in H1334 or H441 cells (Fig 3E), it was at first surprising that there was an increase in nucleotide precursors. However, in H1334 cells this increased occurred later than in A549 cells as it was not observed until 72 hours, and in H441 cells the increase was generally not as large as in A549 cells. Furthermore, it is possible that a block in the conversion of mono- to diphosphorylated-nucleotides occurs in all these cell lines, thus causing an increase in mono-nucleotides.

### NAD depletion results in differential responses to oxidative stress across cell lines

**Fig 5:**
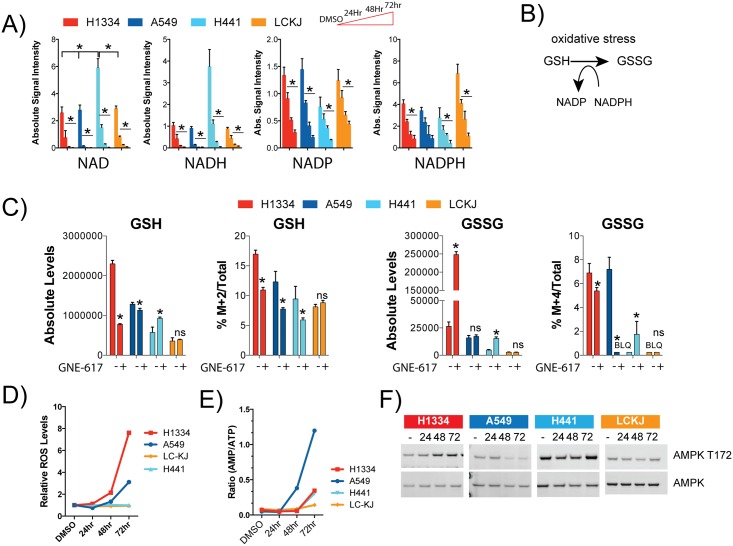
Ability to combat ROS varies across cell lines. NAD, NADH, NADP and NADPH in each cell line were quantitatively determined at 24, 48 and 72 hours following exposure to 0.4 μM GNE-617 (Ave ± SD, n = 5). * indicates a p-value of <0.05, and statistical analysis was performed using a Mann Whitney t-test (two-tailed). B) Schematic showing the cycle of oxidation and reduction of GSG and GSSG. C) Each cell line was exposed to 0.2 μM GNE-617 for 48 hours and labeled with [13C-6]glucose and levels of newly synthesized GSH and GSSG were determined (Ave ± SD, n = 3). * indicates a p-value of <0.05, and statistical analysis was performed using a Mann Whitney t-test (two-tailed); “ns” indicates not-statistically significant. D) ROS levels were determined in each cell line at 24, 48 and 72 hours following exposure to 0.2 μM GNE-617 (shown is the Ave, n = 2). E) Levels of AMP and ATP in each cell line were quantitatively determined at 24, 48 and 72 hours following exposure to 0.4 μM GNE-617 (Ave ± SD, n = 5), and the ratio of the average AMP/ATP levels are shown. F) Western blot analysis showing levels of AMPK and activated AMPK (T172) at 24, 48 and 72 hours following exposure to 0.4 μM GNE-617. Full-length western blots are shown in S2D Fig.

Baseline NAD levels in H1334, A549 and LC-KJ cell lines were very similar, while H441 cells had a ~2-fold higher baseline level (Fig 5A) consistent with these cells having higher NAMPT protein levels (Fig 1F). Associated with the decrease in NAD levels, there was a corresponding decrease in NADH, NADP, and NADPH in all cell lines (Fig 4A).

The ratio of reduced to oxidized glutathione (GSH:GSSG) is another indicator of oxidative stress, as cells accumulate GSSG in response to elevated levels of free radicals (Fig 5B)[21]. Moreover, the reduction of GSSG to GSH requires NADPH, which is predominantly provided by the PPP. Treatment of H1334 cells with GNE-617 for 48 hours does not affect levels of newly synthesized GSSG (Fig 5C). However, an increase in total GSSG levels may be attributed to the inability of these cells to reduce GSSG to GSH due to low NADPH levels. In contrast, production of newly synthesized GSSG is blocked by GNE-617 in A549 cells which resulted in minimal changes in levels of total GSSG. Levels of GSSG in H441 and LC-KJ cells were modestly or not significantly changed with GNE-617 treatment. As LC-KJ cells had higher baseline levels of NADPH (Fig 5A) we hypothesize that they may be better adapted to manage an increase in oxidative stress compared to the three other cell lines. Also, baseline levels of GSH and GSSG were lower in LC-KJ cells, suggesting that these cells may not require high levels of glutathione metabolites under normal growing conditions.

To examine levels of oxidative stress in cells, ROS levels in each cell line were examined at 24, 48 and 72 hours following NAMPT inhibition (Fig 5D). ROS levels were highest in H1334 cells, consistent with these cells increasing GSSG levels after GNE-617 treatment. In contrast, an increase in ROS levels in A549 cells was not observed until 72 hours, and at that time point ROS levels were lower than observed in H1334 cells. No increase in ROS levels were detected in H441 or LC-KJ cells, even though NADPH levels decreased in both cell lines.

### H1334 cells respond to metabolic stress by inducing AMPK

A key metabolic sensor in cells is the enzyme AMP-kinase, which is activated in response to an increase in the AMP/ATP ratio [22]. In A549 cells and to a lesser extent in H1334 and H441 cells, there was an increase in AMP levels and a decrease in ATP levels, which led to an increase in the AMP/ATP ratio at 48–72 hours (Fig 5E). This increase in the AMP/ATP ratio promoted activation of AMPK in H1334 cells but not in A549 cells (Fig 5F). However, lack of activation of AMP in A549 cells was not unexpected, given that this cell line harbors a mutation in LKB1 [23] which is the main kinase responsible for activating AMPK in response to metabolic stress.

### H1334 cells increase glutamine uptake to drive OXPHOS

**Fig 6:**
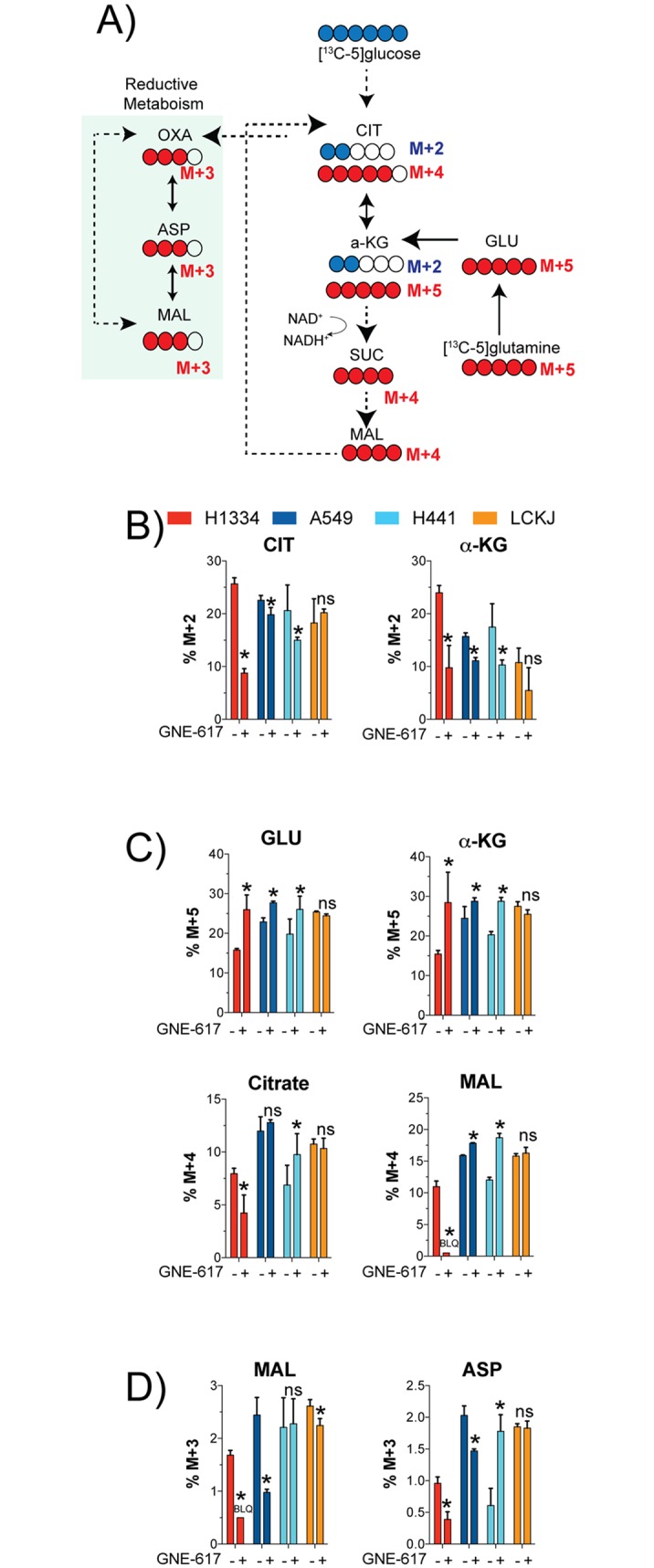
H1334 cells increase OXPHOS in response to NAD depletion. Schematic of carbon flow from glucose or glutamine into OXPHOS. B) Each cell line was exposed to 0.2 μM GNE-617 for 48 hours and labeled with [13C-6]glucose. Levels of newly synthesized citrate (CIT) and α-ketoglutarate (α-KG) were determined (Ave ± SD, n = 3). * indicates a p-value of <0.05, and statistical analysis was performed using a Mann Whitney t-test (two-tailed); “ns” indicates not-statistically significant. C) Each cell line was exposed to 0.2 μM GNE-617 for 48 hours and labeled with [13C-5]glutamine. Levels of newly synthesized glutamate (GLU), α-ketoglutarate (α-KG), citrate (CIT) and malate (MAL) were determined (Ave ± SD, n = 3). * indicates a p-value of <0.05, and statistical analysis was performed using a Mann Whitney t-test (two-tailed); “ns” indicates not-statistically significant. D) Each cell line was exposed to 0.2 μM GNE-617 for 48 hours and labeled with [13C-5]glutamine. Levels of newly synthesized malate (MAL) and aspartate (ASP) generated by reductive metabolism (quantify the M+3 isotopomers) were determined (Ave ± SD, n = 3). * indicates a p-value of <0.05, and statistical analysis was performed using a Mann Whitney t-test (two-tailed); “ns” indicates not-statistically significant.

Given the large effects of NAD depletion on global metabolic pathways (Fig 2A), we also examined how oxidative phosphorylation (OXPHOS) was modulated across cell lines (Fig 6A). To assess carbon flow from glycolysis into OXPHOS, cells were labeled with [^13^C-6]-glucose and levels of metabolites associated with OXPHOS were examined (Fig 6B). In H1334 cells, and to a lesser extent in A549 and H441 cells, there was a reduction in the M+2 isotopomer of citrate, indicating that these cells reduced carbon flow from glycolysis into OXPHOS. This is consistent with reduced levels of glycolytic intermediates such as pyruvate that were observed in these cell lines (Fig 3B). Thus, all three of these cell lines reduced carbon flow from glycolysis into the OXPHOS to varying levels.

Cells can increase glutamine uptake to compensate for reduced glycolysis and still drive OXPHOS. We assessed the contribution of glutamine to OXPHOS by labeling cells with [^13^C-5]-glutamine and measuring levels of newly synthesized metabolites (Fig 6A). All three sensitive cell lines increased glutamine (%M+5 isotopomer) to varying levels (Fig 6C). In contrast to A549 and H441 cells, H1334 cells reduced carbon flux to malate and citrate (Fig 6C), indicating that these cells have dramatically reduced their ability to utilize the TCA cycle. Thus, both A549 and H441 cell lines attempt to compensate for reduced glycolytic capacity by modestly up-regulating OXPHOS through glutamine uptake.

Finally, cells can also use reductive glutamine metabolism (Fig 6A) to generate NADPH for driving fatty acid synthesis. Both sensitive cell lines, A549 and H1334, decreased the M+3 isotopomer of malate and aspartate, whereas effects in H441 cells were minimal (decrease of malate and an increase of aspartic acid) (Fig 6D). LC-KJ cells did not show any change in levels of these metabolites. Thus, cells do not appear to consistently utilize reductive glutamine metabolism in response to loss of NAD.

## Discussion

Here we examine the underlying consequences of NAD depletion with the NAMPT inhibitor GNE-617 on global metabolism in four different NSCLC cell lines. Even though there are published reports of metabolic profiling following NAMPT inhibition, there is no systematic study that compares the effects of NAD depletion across sensitive and resistant cell lines. We show that the effects of NAD depletion are surprisingly divergent across NSCLC cell lines and we propose that some of these differences may be driven by genetic variability.

Metabolic profiling was undertaken with two cell lines sensitive to NAMPT inhibition (H1334 and A549), one cell line that showed intermediate sensitivity (H441) and one cell line that was inherently insensitive in short term viability assays (LC-KJ). It was previously reported that depletion of NAD reduced glycolytic flux [10, 12, 20] and, consistent with this, all three sensitive cell lines displayed, to varying degrees, an increase in early glycolytic intermediates and a reduction in downstream glycolytic metabolites, consistent with reduced activity of GAPDH, an NAD utilizing enzyme (Fig 3A).

A consequence of a block in glycolysis is that cells can divert carbon flow into the PPP, which generates R-5-P for the biosynthesis of purines and pyrimidines as well as NADPH for the synthesis of fatty acids, cholesterol and reduced glutathione (Fig 3C). NADPH is a key metabolite that is used by cells to combat oxidative stress, which can be quantified by ROS levels and by the ratio of reduced to oxidized glutathione (GSG:GSSG ratio). Given the large changes in global metabolism, it is not surprising that ROS levels increased in both A549 and H1334 cell lines following NAD depletion (Fig 5D). Unlike H1334 cells, A549 cells increased flux through the PPP (Fig 3C-3D), which could be an attempt to combat raising levels of ROS. Consistent with this, A549 cells have lower ROS levels than H1334 cells (Fig 5D) and A549 cells do not show an increase in GSSG levels (Fig 5C), suggesting that they can still combat oxidative stress. The ability of A549 cells to combat oxidative stress may be partly attributed to the fact that these cells harbor a homozygous mutation in KEAP1, which results in constitutive activation of NRF2, a transcription factor that drives expression of proteins encoding cellular antioxidants [24].

Surprisingly, H441 cells did not display an increase in ROS levels, even though there was a large decrease in NAD, NADH, NADP and NADPH levels (Fig 5A). One possible reason is that these cells have higher baseline levels of NAMPT protein and higher baseline levels of NAD and NADH. Notably the baseline levels of glutathione in A549, H1334, and H441 cells were all higher than in LC-KJ cells (Fig 5D), which may also suggest that these cells have higher basal levels of oxidative stress and thus may be more susceptible to the effects of NAD depletion compared to LC-KJ cells.

Cell lines that were sensitive to NAMPT inhibition also had a significant reduction in di- and tri-phosphorylated nucleotides, and this was associated with a corresponding increase in levels of mono-phosphorylated nucleotides (Fig 4C). One important consequence of an increase in mono-phosphorylated nucleotides is that the ratio of AMP to ATP will increase, which is an important sensor of metabolic stress. As this ratio increases, AMPK is activated, stimulates pathways involved in catabolism to increase ATP levels and inhibits pathways that consume ATP [22]. H1334 cells activate AMPK in response to an increase in the AMP/ATP ratio whereas H441 cells only show a modest increase in activated AMPK, possibly due to these cells having higher baseline lines of phosphorylated T172-AMPK than in the other cell lines. Thus, higher baseline levels of AMPK may help sustain a low AMP/ATP ratio. Even though A549 cells increase their AMP/ATP ratio, they do not activate AMPK due to a mutation in LKB, a key upstream kinase that phosphorylates and activates AMPK [23].

In contrast to GNE-617 sensitive cell lines, minimal metabolic effects were noted in LC-KJ cells, which were the most refractory to NAD depletion. There are a number of reasons that may explain differential sensitivities across these four cell lines to NAMPT inhibition. Both of the two most sensitive cell lines (H1334 and A549) have a similar doubling time, similar levels of NAMPT protein, and also similar NAD baseline levels (Table 1). However, what differentiates these two cell lines from each other is that A549 can rapidly utilize the PPP and has lower levels of ROS after NAMPT inhibition, suggesting that their ability to increase the PPP helps them combat an increase in ROS levels. In contrast, H1334 cells do not have the capacity to increase flux through the PPP to combat rising ROS levels, which may explain their enhanced sensitivity to NAMPT inhibition. The less sensitive cell line, H441, has higher baseline level of NAMPT protein and NAD levels which alone may make them less prone to the immediate effects of NAD depletion. Finally, the one differentiating factor that distinguishes LC-KJ cells, which are relatively insensitive cell line, is that it has a noticeably longer doubling time. Also, these cells have a higher baseline level of NADPH, which may increase their ability to combat oxidative stress due to loss of NAD. Thus, reduced sensitivity of LC-KJ cells may be attributed to their lower metabolic requirement due to a slow doubling time and to enhanced NADPH reservoirs to combat cellular stress.

**Table 1:** Summary of cell line characterization (see text for details).

	H1334	A549	H441	LC-KJ
GNE-617 Viability EC_50_(nM)*	1.4	18.9	3370	>5000
Cell doubling time (hrs)	35	32	43	**65**
Relative NAMPT protein	1	1.22	**2.23**	1.08
Relative NAD baseline Levels	1	1.07	**2.27**	1.12
Relative NADPH baseline levels	1	1.32	1.07	**2.63**
Increase of PPP after NAMPT inhibition	No	**Yes**	No	No
ROS levels after NAMPT inhibition	**High**	Medium	No increase	No increase
AMPK activation after NAMPT inhibition	Yes	No	No	No

* Viability determined in 96-hour assay. See Figs 1B and S1

While NAMPT levels correlate with sensitivity to NAMPT inhibition, our data suggests that predicting sensitivity is more complicated. For example, LC-KJ cells have similar NAMPT levels as the sensitive cell lines A549 and H1334, yet they are inherently more resistant to NAMPT inhibition. We hypothesize that the underlying metabolic rate of a cell, or tumor, may be an important contributor to sensitivity, as a cell line with a slow doubling time may have a low metabolic turnover and thus be more resistant to NAD depletion. Additionally, our data demonstrates that the underlying profile of each cell line can modulate the metabolic response to NAMPT inhibition. This raises the possibility that inhibitors targeting different metabolic pathways may be effectively combined with a NAMPT inhibitor in some cell lines; however, these combinations may have to be tailored to match the underlying genetic and metabolic dependency of a particular cell. Nevertheless, our data suggests that novel combinations may be used to maximize the benefit of a NAMPT inhibitor.

This study revealed striking differences in the response of different cell lines to NAD depletion. While the two sensitive cell lines showed defects in glycolysis, a decrease in di-and tri-phosphorylated nucleotides, and a modest increase in OXPHOS, they coped with increased metabolic stress differently. H1334 cells activated AMPK whereas A549 cells, which have a mutation in LKB1, were unable to activate AMPK. However, A549 cells dramatically increased carbon flux through the PPP, unlike H1334 cells, and contain a mutation in KEAP1 that results in constitutive activity of NRF2. Thus, even though these cell lines are similarly sensitive to NAMP inhibition, their metabolic response can be governed by their underlying genetic profile and metabolic dependencies. In conclusion, our data reveal that depletion of a single key central metabolite such as NAD can have wide effects on overall cellular metabolism and that each cell line has a unique way of dealing with metabolic stress that can be dictated by its genetic profile.

## Supporting Information

S1 Fig NAMPT protein levels and sensitivity to GNE-617. **A)** Western blot analysis of NAMPT protein levels across a panel of NSCLC cell lines. **B)** Shown is the IC_50_ value for GNE-617 for each of the 4 cell lines used in this study. Cells were incubated with a dose response of GNE-617 for 4 days (n = 3, ± SD).
(TIF)

S2 Fig Larger images of gels. **A)** Larger image of western blot shown in Fig 1F. Dashed box indicates the cropped area shown in final figure. **B)** Larger image of western blot shown in Fig 3F. Dashed box indicates the cropped area shown in final figure. **C)** Larger image of western blots shown in Fig 5F. Dashed box indicates the cropped area shown in the final figure. **D)** Larger images of gels shown in S1 Fig. The upper two panels are the upper gels in S1 Fig, and the lower two gels are the low gels in S1 Fig. In each case the gels were first probed to detect NAMPT protein levels, and then immediately re-probed to detect Actin protein levels.
(TIF)

S1 Table Metabolic profiling of H1334, A549, H441 and LC-KJ cells treated with GNE-617 for 24, 48 or 72 hours. The raw data for each metabolite at each time-point is shown (n = 5), along with the average data and the log-_2_ fold change for each metabolite relative to the level determined in control (DMSO) treated cells.
(XLSX)
