# Supplementary figures and images for: Metabolic Response to NAD Depletion across Cell Lines Is Highly Variable
Source: PLoS One. 2016 Oct 6;11(10):e0164166. doi: 10.1371/journal.pone.0164166 (PMC5053472; doi:10.1371/journal.pone.0164166)

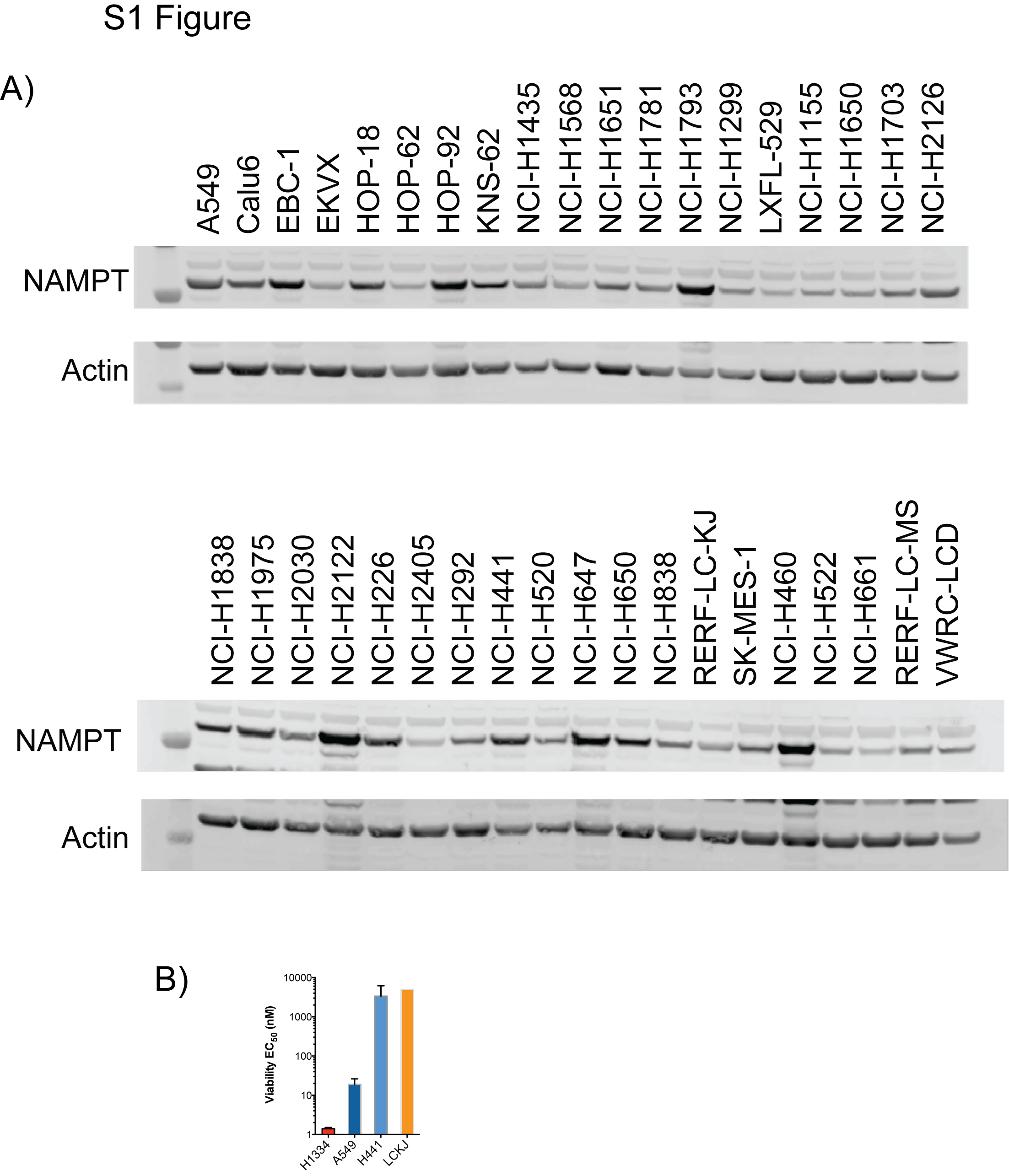

Supplement: S1 Fig — A) Western blot analysis of NAMPT protein levels across a panel of NSCLC cell lines. B) Shown is the IC50 value for GNE-617 for each of the 4 cell lines used in this study. Cells were incubated with a dose response of GNE-617 for 4 days (n = 3, ± SD). (TIF) [file pone.0164166.s001.tif]

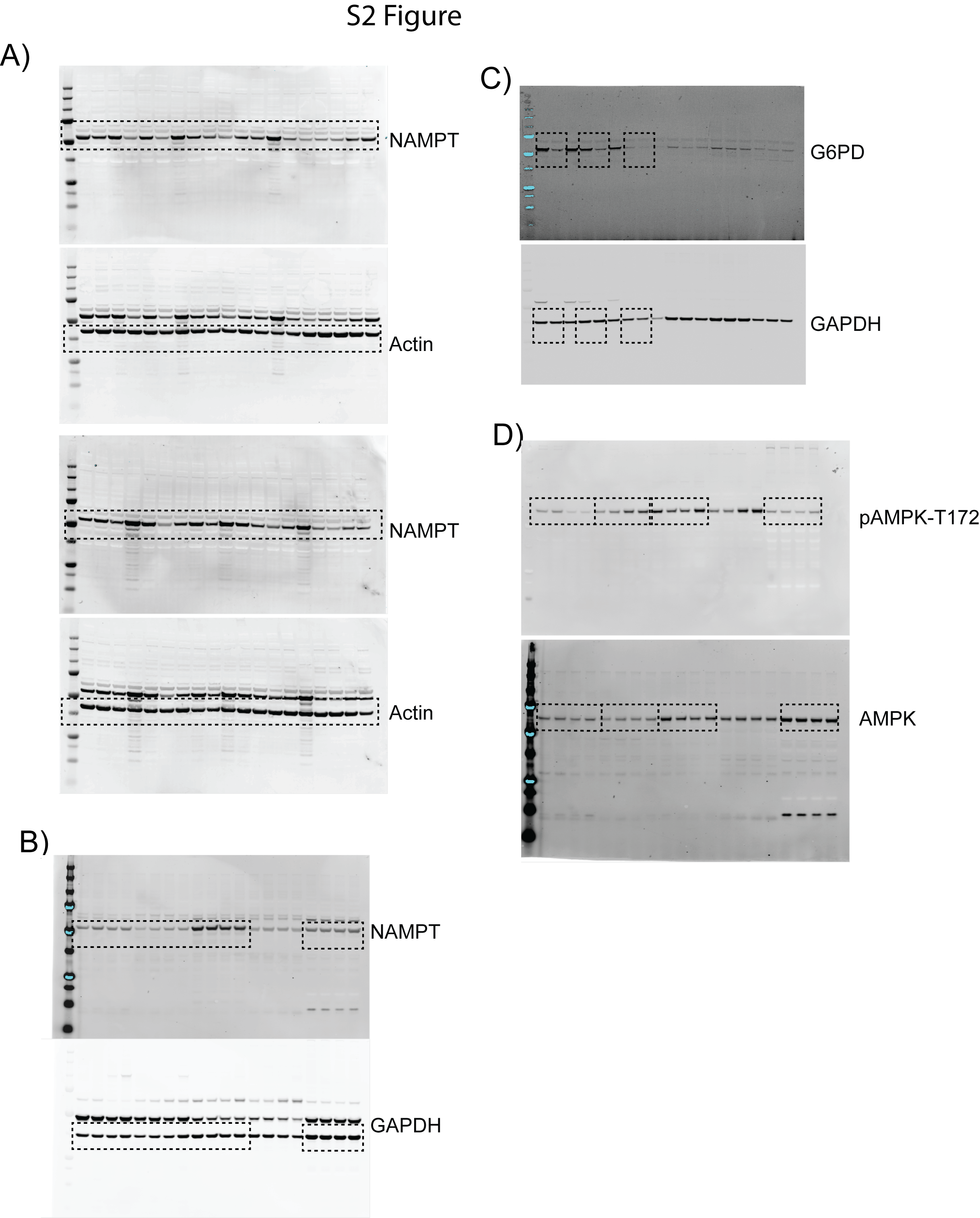

Supplement: S2 Fig — A) Larger image of western blot shown in Fig 1F. Dashed box indicates the cropped area shown in final figure. B) Larger image of western blot shown in Fig 3F. Dashed box indicates the cropped area shown in final figure. C) Larger image of western blots shown in Fig 5F. Dashed box indicates the cropped area shown in the final figure. D) Larger images of gels shown in S1 Fig. The upper two panels are the upper gels in S1 Fig, and the lower two gels are the low gels in S1 Fig. In each case the gels were first probed to detect NAMPT protein levels, and then immediately re-probed to detect Actin protein levels. (TIF) [file pone.0164166.s002.tif]
